# Maximum Entropy Principle Based Estimation of Performance Distribution in Queueing Theory

**DOI:** 10.1371/journal.pone.0106965

**Published:** 2014-09-10

**Authors:** Dayi He, Ran Li, Qi Huang, Ping Lei

**Affiliations:** School of Humanities & Economic Management, Lab of Resources & Environment Management, China University of Geosciences (Beijing), Beijing, P. R. China; University of Zurich, Switzerland

## Abstract

In related research on queuing systems, in order to determine the system state, there is a widespread practice to assume that the system is stable and that distributions of the customer arrival ratio and service ratio are known information. In this study, the queuing system is looked at as a black box without any assumptions on the distribution of the arrival and service ratios and only keeping the assumption on the stability of the queuing system. By applying the principle of maximum entropy, the performance distribution of queuing systems is derived from some easily accessible indexes, such as the capacity of the system, the mean number of customers in the system, and the mean utilization of the servers. Some special cases are modeled and their performance distributions are derived. Using the chi-square goodness of fit test, the accuracy and generality for practical purposes of the principle of maximum entropy approach is demonstrated.

## Introduction

**Figure 1:**
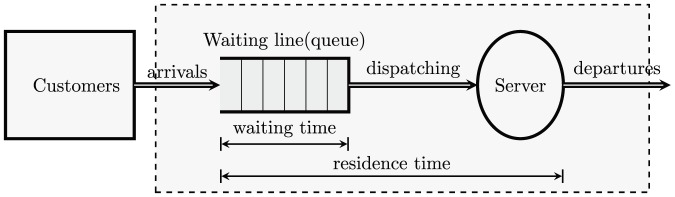
Queuing System Structure with Single-Server.

Queuing theory is mainly regarded as a branch of applied probability theory. Its applications are in different fields, such as communication networks, computer systems, machine plants, and services. Fig. 1 is a typical queuing system with a single server.

Queuing theory tries to answer questions like the mean waiting time in the queue, the mean system response time (waiting time in the queue plus service time), the mean utilization of the service facility, the distribution of the number of customers in the queue, and the distribution of the number of customers in the system. These questions are mainly investigated in a stochastic scenario, where, for example, the inter-arrival times of the customers or the serving times are assumed to be random typically Poisson arrivals and to have exponent distribution serving times.

**Figure 2:**
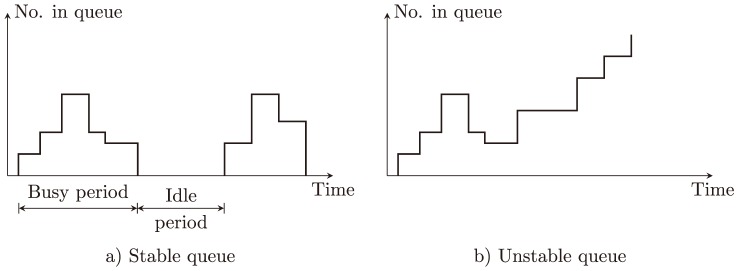
Stable and Unstable Queueing System.

However, usually we are mainly interested in steady state solutions (see Figure 2); that is, where the system after a long running time tends to reach a stable state in which, for example, the distribution of customers in the system does not change (limiting distribution).

In a canonical way, the steady state of system performance can be derived from assumptions on the distribution of inter-arrival times and service times. Hence, these assumptions are the basic requirement for analyzing queuing systems. However, in practical situations the pre-assumed distributions are difficult to satisfy or to acquire. To some degree, this fact limits the practical applications of queuing theory.

The maximum entropy principle is applicable to queuing theory because very often only partial information is available about the probability distributions. With respect to queuing theory, the principle of maximum entropy has been applied to solving numerous systems including, but not limited to, 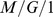 and 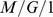 queues ([1], [2]), finite and infinite capacity 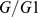 queues ([3], [4]), multi-server queues ([5], [6]), multiple class queues with priorities ([7]), and queues with vacation ([8]–[10]) and queuing networks ([11]–[13]). In fact, since the early 1970's many attempts have been made to apply the method of maximum entropy in the field of queuing theory. Ferdinand [14] used the method to derive the equilibrium solution of the 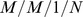 system by analogy with statistical mechanics. Shore [15] built an abstract model from which he determined the maximum entropy solution of the 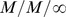 and 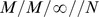 systems. Bard [16] applied entropy maximization to a class of problems in the performance evaluation of computer systems. El-Affendi and Kouvatsos [2] used the maximum entropy principle to analyze the 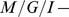 and 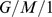-queuing systems at equilibrium. Alfa and Chen [17] developed a discrete time approach model for obtaining the expected queue length of the 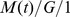 queue. Arizono, Cui, and Ohta [18] analyzed 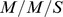 using the maximum entropy principle. Falin, Martin, and Artalejo [19] presented information on theoretic approximations for the 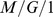 queue with retrials. Kouvatsos and Tabet-Aouel [20] applied entropy maximization to characterize the distributional form of the steady-state probabilities of a 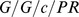 queue with 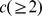 parallel servers and 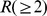 priority classes under a pre-emptive resume (PR) rule. Tadj and Hamdi [21] dealt with a quorum queuing system with a threshold level 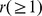 that regulates the beginning and ending of idle and busy periods as follows: an idle period starts when the queue size drops below level 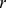 and a busy period starts as soon as the queue accumulates the same number 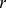. The single server processes 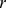 customers in one batch. They denoted this system as 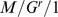 by Kendall's notation. They presented a maximum entropy model to determine the distribution of related random variables with known server utilization and mean queue length.

The work mentioned above applied the principle of maximum entropy to certain kinds of queuing systems based on assumptions concerning inter-arrivals and server times, which limited the practical applications of queuing theory. In fact, if a queue system is stable, this is not necessary when the maximum entropy method is applied to a certain queuing system. In this study, we consider the queuing system as a black box and derive a performance index for the queuing system by the principle of maximum entropy only on the assumption that the queue is stable instead of making assumptions on the distribution of inter-arrival times and service times. Meanwhile, from the viewpoint of expanding the practical application of queuing systems, we use some easily accessible indexes of queuing systems, such as the capacity of the system, the mean of customers in the queue, and the mean utilization of the system. Based on these indexes and the principle of maximum entropy, optimization models are then developed to derive the performance of queuing systems.

This paper is organized as follows. Section 2 is a simple review of the maximum entropy principle. Sections 3 and 4 develop different maximum entropy models with known mean numbers of customers and the average value of busy periods under unlimited and limited server capacity, respectively. Section 5 compares our results with general models with known assumptions on the distributions of inter-arrival times and server times by the 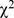 goodness of fit test. The last section concludes.

## Methods: The Principle of Maximum Entropy

The principle of maximum entropy provides a solution to the old problem of the assignment of a probability distribution to a random variable that avoids bias while satisfying given or known information about the random variable. Jaynes is credited with having formalized the principle of maximum entropy in [22].

Mathematically the principle can be presented as follows: consider a system 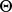 that has a finite or countable infinite set 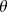 of possible states 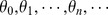. Let 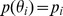 be the probability that the system 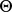 is in state 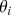. Suppose all that is known about these probabilities are 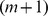 constraints of the form
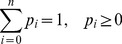
(1)

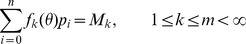
(2)where 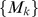 are expectations defined on a set of suitable functions 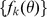, which can be looked at as the known information. Since, in general, the number of constraints is less than the number of possible states, one is faced with an infinite number of distributions 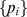 that satisfy these constraints. The problem is which one to choose.

The maximum entropy principle states that, of all the distributions satisfying the constraints supplied by the given information, the minimally prejudiced distribution that should be chosen is the one that maximizes the system entropy,



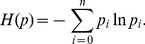
(3)


To sum up, the maximum entropy principle can be described as the following model for discrete variants.



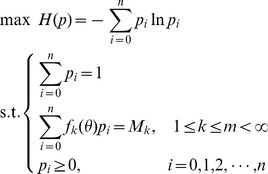
(4)


This is a natural extension of Laplace's famous principle of insufficient reason, which postulates that the uniform distribution is the most satisfactory representation of our knowledge when we know nothing about the random variable except that each probability is non-negative and that the sum of the probabilities is unity.

The maximization of (3) subject to constraints (1) and (2) can be solved by using the Lagrangian method of undetermined multipliers leading to the solution:



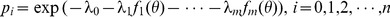
(5)where 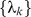 are the Lagrangian multipliers corresponding to the set of constraints (1) and (2). More details on the maximum entropy principle and its generalizations can be found in [23], [24].

Especially, if only the expected value 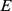 is known, the second constraint converts to



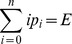
and according to (5) the estimated distribution is



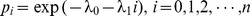



To be simple, let



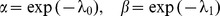
then



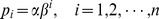



Substituting the above equation into the constraints, we can get



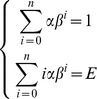



Hence,



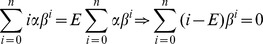
where the coefficients of 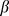 in the above equation are increasing, and 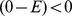 and there exists an 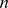 that makes 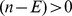, so that there is only one change in sign in the coefficients of 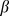. According to Descartes's rule of signs, there is only one positive real root to the above equation. This indicates the solution uniqueness of the maximum entropy estimation problem even if there are only expected value and unit and non-negative requirements, which provides the basis for the later study in this article.

The maximum entropy approach to queuing systems is based on finding a maximum-entropy performance distribution based on the knowledge of some moments of the distribution concerned. To simplify, we will only discuss a queuing system with a single server and infinite customers, and where the dispatching rule is FIFO (First In First Out). The queuing systems are classified by server capacity into two types: queuing systems with either unlimited or limited server capacity. In each type, we will estimate the distribution of the system state by the maximum entropy principle from the mean number of customers in the system and the average value of a busy period.

## Discussion

### 1. Queuing system with unlimited server capacity

If it has unlimited server capacity, a queuing system can serve as many customers as possible. Generally speaking, it is hard to discover the distributions of customer arrivals and departures. Hence, for a queuing system it is easy and practical to acquire knowledge of the mean number of customers and the busy periods etc. if the queue is stable.

Let the mean number of customers in the system under steady state be 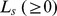, and 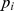 is the probability of the fact that there are 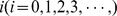 customers in the queuing system. According to the maximum entropy principle, the following model can be established if there is no more information.



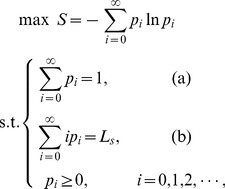
(6)


Based on the method in Section 2, the distribution of the system state is



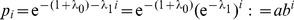
(7)with (6.a) and (6.b),



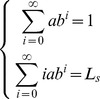
(8)As we know, the above equations have only one positive real root. As for 
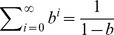
 (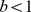), we can get



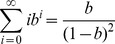
so with (8)



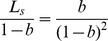
then,



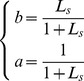
(9)so



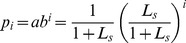
(10)Let



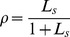
(11)then



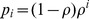
(12)The results in (12) reflect the probability distribution function of a single server queuing system with a known mean number of customers and without limitation on system capacity, from which we can obtain the performance indexes of this kind of queuing system. It should be noted that the results presented here are coherent with a 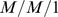 queuing system. The intrinsic reason is that the maximum entropy distribution with a known non-negative mean value is a Poisson distribution [25].

Another situation is that the queuing system has unlimited server capacity and a known mean server utilization of of 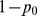. Under this situation, the maximum entropy model is changed to be:



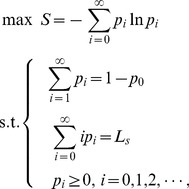
(13)


With a similar approach, we can get



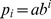
(14)where
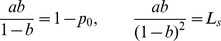
(15)that makes
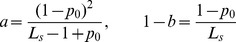
(16)then



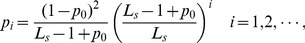
(17)Then, with a known 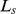 and 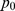, the system performance can be achieved.

### 2. Queuing system with limited server capacity

In this section we will study the situation where the queuing system has a limited capacity; that is, there are 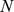 customers at most in the system, 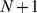 customers will leave, and the other assumptions are the same as before.

Firstly, we will consider the queuing system with only a known mean customer 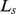. Then, the maximum entropy model is:
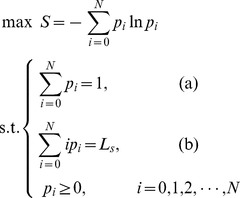
(18)


By a similar method, we come to
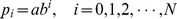
(19)where 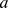 and 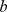 are the roots of the following equations



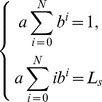
(20)As we know, the above equations have only one positive root. And we define
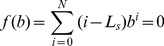
(21)


And because
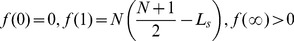
(22)we can come to the following results:

- if 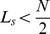, then 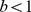,
- if 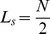, then 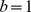 and the distribution of steady state is an average distribution,
- if 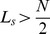, then 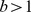.

Table 1 and Table 2 demonstrate the above results. In Table 1 the known mean number of customers is assumed to be 6 and in Table 2 the number is assumed to be 8. In Table 1, the capacity of the system is set to be 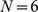, when 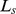 is set to be different values, we can get 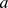
' and 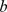
'value by solving (20). Then, by using (19) the value of 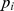 can be calculated. Then, the maximum entropy can be calculated too. By similar approach, Table 2 can be achieved.

**Table 1:** Maximum Entropy Distribution (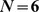).

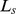	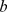	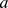	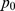	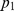	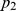	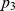	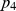	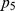	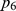	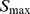
0.5	0.335	0.666	0.666	0.223	0.075	0.025	0.008	0.003	0.001	0.954
1.0	0.517	0.488	0.488	0.252	0.130	0.067	0.035	0.018	0.009	1.378
1.5	0.651	0.367	0.367	0.239	0.156	0.101	0.066	0.043	0.028	1.646
2.0	0.768	0.276	0.276	0.212	0.162	0.125	0.096	0.074	0.057	1.818
2.5	0.881	0.202	0.202	0.178	0.157	0.138	0.122	0.107	0.095	1.915
**3.0**	**1.000**	**0.143**	**0.143**	**0.143**	**0.143**	**0.143**	**0.143**	**0.143**	**0.143**	**1.946**
3.5	1.135	0.095	0.095	0.107	0.122	0.138	0.157	0.178	0.202	1.915
4.0	1.302	0.057	0.057	0.074	0.096	0.125	0.162	0.212	0.276	1.818
4.5	1.536	0.028	0.028	0.043	0.066	0.101	0.156	0.239	0.367	1.646
5.0	1.935	0.009	0.009	0.018	0.035	0.067	0.130	0.252	0.488	1.378
5.5	2.987	0.001	0.001	0.003	0.008	0.025	0.075	0.223	0.666	0.954

**Table 2:** Maximum Entropy Distribution (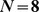).

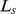	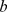	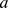	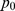	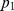	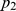	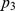	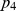	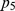	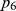	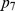	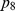	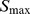
0.5	0.334	0.667	0.667	0.222	0.074	0.025	0.008	0.003	0.001	0.000	0.000	0.955
1.0	0.505	0.496	0.496	0.251	0.126	0.064	0.032	0.016	0.008	0.004	0.002	1.384
1.5	0.618	0.387	0.387	0.239	0.148	0.092	0.057	0.035	0.022	0.013	0.008	1.671
2.0	0.707	0.306	0.306	0.217	0.153	0.108	0.077	0.054	0.038	0.027	0.019	1.876
2.5	0.785	0.243	0.243	0.190	0.149	0.117	0.092	0.072	0.057	0.044	0.035	2.022
3.0	0.857	0.191	0.191	0.163	0.140	0.120	0.103	0.088	0.075	0.065	0.055	2.121
3.5	0.927	0.148	0.148	0.137	0.127	0.118	0.109	0.101	0.094	0.087	0.081	2.178
**4.0**	**1.000**	**0.111**	**0.111**	**0.111**	**0.111**	**0.111**	**0.111**	**0.111**	**0.111**	**0.111**	**0.111**	**2.197**
4.5	1.079	0.081	0.081	0.087	0.094	0.101	0.109	0.118	0.127	0.137	0.148	2.178
5.0	1.168	0.055	0.055	0.065	0.075	0.088	0.103	0.120	0.140	0.163	0.191	2.121
5.5	1.275	0.035	0.035	0.044	0.057	0.072	0.092	0.117	0.149	0.190	0.243	2.022
6.0	1.414	0.019	0.019	0.027	0.038	0.054	0.077	0.108	0.153	0.217	0.306	1.876
6.5	1.617	0.008	0.008	0.013	0.022	0.035	0.057	0.092	0.148	0.239	0.387	1.671
7.0	1.981	0.002	0.002	0.004	0.008	0.016	0.032	0.064	0.126	0.251	0.496	1.384
7.5	2.998	0.000	0.000	0.000	0.001	0.003	0.008	0.025	0.074	0.222	0.667	0.955

If the system with limited server capacity has information of mean customer number 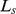 and busy period 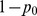, the maximum entropy model will be
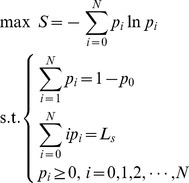
(23)


So we get
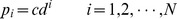
(24)where
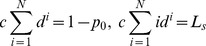
(25)that is



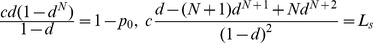
(26)The solution is the root of the following equation:
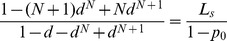
(27)


By equation (27) with known 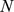, 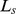, and 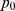, the value of 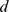 can be decided and then 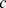 can be decided too by equation (26): then we can get the steady state distribution 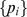. Similar results can be derived:

- if 
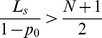
, then 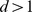,
- if 
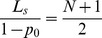
, then 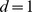,
- if 
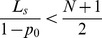
, then 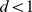.

In Tables 3 and 4, we assume 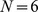 and 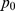 equals 0.1 and 0.01, respectively; then we calculate the system performance distribution by changing the value of 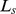. In Table 3, by using (26) the value of 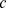 and 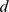 can be got. Then substituting 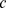 and 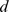 into (24) the value of 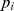 can be calculated and the corresponding entropy can be calculated too. In similar procedure, we can get Table 4.

**Table 3:** Maximum Entropy Distribution (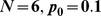).

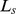	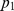	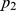	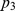	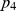	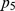	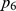	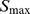
1.00	0.810	0.081	0.008	0.001	0.000	0.000	0.650
1.50	0.533	0.219	0.090	0.037	0.015	0.006	1.331
2.00	0.374	0.226	0.137	0.083	0.050	0.030	1.669
2.50	0.260	0.201	0.155	0.120	0.093	0.072	1.856
3.00	0.172	0.163	0.154	0.145	0.137	0.129	1.933
**3.15**	**0.150**	**0.150**	**0.150**	**0.150**	**0.150**	**0.150**	**1.938**
3.50	0.104	0.119	0.137	0.156	0.179	0.205	1.914
4.00	0.053	0.075	0.106	0.151	0.213	0.302	1.795
4.50	0.018	0.035	0.065	0.122	0.229	0.430	1.556
5.00	0.002	0.006	0.019	0.060	0.193	0.621	1.127

**Table 4:** Maximum Entropy Distribution (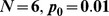).

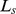	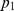	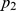	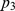	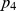	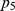	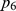	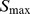
1.000	0.980	0.010	0.000	0.000	0.000	0.000	0.112
1.500	0.650	0.224	0.077	0.027	0.009	0.003	1.016
2.000	0.467	0.252	0.136	0.073	0.040	0.021	1.422
2.500	0.338	0.236	0.165	0.115	0.080	0.056	1.663
3.000	0.239	0.203	0.172	0.146	0.124	0.105	1.792
3.500	0.160	0.162	0.164	0.166	0.168	0.170	1.830
**3.465**	**0.165**	**0.165**	**0.165**	**0.165**	**0.165**	**0.165**	**1.830**
4.000	0.098	0.118	0.143	0.172	0.208	0.252	1.780
4.500	0.050	0.074	0.110	0.162	0.240	0.354	1.636
5.000	0.018	0.035	0.067	0.130	0.252	0.489	1.377
5.500	0.002	0.006	0.020	0.066	0.212	0.683	0.938

### 3. The chi-square goodness of fit test

Without any assumptions on the distribution of inter-arrival times and server times, we deduced the performance distributions of the queuing system by the maximum entropy principle above. Is this method effective and feasible? We will test our method by the 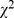 goodness of fit test to determine this.

Taking model (18) as an example, if we know the distribution of inter-arrival times and server times follow the Poisson process, then we get a 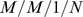 queuing system. Its performance distribution is
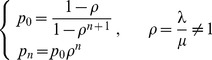
(28)and the mean number of customers in the system is



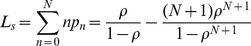
(29)We can decide the value of 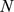 and 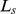 and get the value of 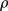 by solving (29), the theoretic distribution can be calculated by using (28). The results are shown in Table 5.

**Table 5:** Theoretic Distribution of 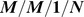 (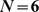).

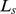	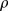	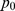	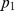	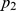	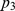	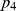	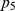	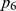
0.5	0.335	0.665	0.223	0.075	0.025	0.008	0.003	0.001
1.0	0.517	0.488	0.252	0.130	0.067	0.035	0.018	0.009
1.5	0.651	0.367	0.239	0.156	0.101	0.066	0.043	0.028
2.0	0.768	0.276	0.212	0.162	0.125	0.096	0.074	0.057
2.5	0.881	0.202	0.178	0.157	0.138	0.122	0.107	0.095
**3.0**	**1.000**	**0.143**	**0.143**	**0.143**	**0.143**	**0.143**	**0.143**	**0.143**
3.5	1.135	0.095	0.107	0.122	0.138	0.157	0.178	0.202
4.0	1.302	0.057	0.074	0.096	0.125	0.162	0.212	0.276
4.5	1.536	0.028	0.043	0.066	0.101	0.156	0.239	0.367
5.0	1.934	0.009	0.018	0.035	0.067	0.130	0.252	0.488
5.5	2.984	0.001	0.003	0.008	0.025	0.075	0.223	0.665

On the other side, if we only know 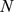 and 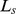 of the queuing system, according to (18) we can arrive at the maximum entropy distribution as shown in Table 1. Comparing Table 5 with Table 1, it can be found that the maximum entropy distribution is very close to the theoretic distribution; that is, the 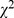 is almost equal to 0. Hence, the maximum entropy distribution is a good estimation.

## Conclusions

Queuing system analysis is usually based on some assumptions about the distributions of inter-arrival times and server times. This study shows that there is no need to assume those distributions, and if a queuing system is looked at as a black box, the system performance can be estimated by the maximum entropy principle with some easily accessible macro-level indexes. In this paper, some common queuing system are studied including queuing system with unlimited server capacity and queuing system with limited server capacity. By utilizing the principle of maximum entropy, and with known information of some easily accessible macro-level indexes such as mean number of customers in the system 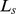, system capacity 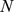 and mean server utilization of of 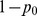, we demonstrate that maximum entropy method is a feasible and effective approach to estimate the system performance distribution.

However, our study focused on single server queuing systems. For further research, multi-server queuing systems should be taken into consideration. For multi-server queuing system, more factors, for example queuing rules, server layout, system capacity et.al., should be considered. Hence, it will be more complicated. However, with assuming on those factors and observed indexes as presented in this paper, we can that our methods will be applicable in those circumstances also.
